# Retrospective comparison of Kahook Dual Blade Excisional Goniotomy with trabecular aspiration in pseudoexfoliation glaucoma at the time of cataract surgery using propensity score matching

**DOI:** 10.1177/25158414251405351

**Published:** 2026-01-06

**Authors:** Alicja Strzalkowska, Piotr Strzalkowski, Alexandra V. Schilcher, Jennifer Prues-Hoelscher, Kristina Spaniol, Gerd Geerling

**Affiliations:** Department of Ophthalmology, Medical Faculty and University Hospital Düsseldorf, Heinrich Heine University Düsseldorf, Moorenstreet 5, Düsseldorf 40225, Germany; Department of Ophthalmology, Medical Faculty and University Hospital Düsseldorf, Heinrich Heine University Düsseldorf, Düsseldorf, Germany; Department of Ophthalmology, Medical Faculty and University Hospital Düsseldorf, Heinrich Heine University Düsseldorf, Düsseldorf, Germany; Department of Ophthalmology, Medical Faculty and University Hospital Düsseldorf, Heinrich Heine University Düsseldorf, Düsseldorf, Germany; Department of Ophthalmology, Medical Faculty and University Hospital Düsseldorf, Heinrich Heine University Düsseldorf, Düsseldorf, Germany; Department of Ophthalmology, Medical Faculty and University Hospital Düsseldorf, Heinrich Heine University Düsseldorf, Düsseldorf, Germany

**Keywords:** goniotomy, Kahook Dual Blade, PEX glaucoma, trabecular aspiration

## Abstract

**Background:**

Minimally invasive glaucoma surgeries (MIGS) have gained attention for their safety and efficacy, especially in clinical trials involving primary open-angle glaucoma. However, real-world data on pseudoexfoliation glaucoma (PEX glaucoma) remain limited. To our knowledge, this is the first real-world study comparing postoperative outcomes of Kahook Dual Blade Excisional Goniotomy (KDB) or trabecular aspiration (TA) in PEX glaucoma.

**Objective:**

To compare the efficacy and safety of combined clear cornea cataract extraction with either KDB or TA in patients with PEX glaucoma.

**Design:**

A retrospective analysis.

**Methods:**

We reviewed 113 eyes from 99 patients who underwent cataract surgery combined with either KDB (*n* = 71) or TA (*n* = 42) between May 2017 and May 2023 at the University Eye Hospital. Propensity score matching was used to create comparable groups of 30 KDB and 30 TA cases based on age, sex, baseline intraocular pressure (IOP), and number of glaucoma medications. Key outcomes included IOP, number of glaucoma medications, complications, and reoperations.

**Results:**

The mean patient age was 80.5 ± 7.3 years for KDB and 80.4 ± 7.7 years for TA, *p* = 0.94. 73.3% were women, *p* = 1.0. The mean baseline IOP was 20.4 ± 7.1 for KDB and 20.0 ± 8.4 for TA, *p* = 0.35, and the baseline number of glaucoma medications (Meds) was 2.0 ± 1.2 for KDB and 2.0 ± 1.2 for TA, *p* = 0.78. The mean postoperative IOP at 12 months was 13.5 ± 3.6 mmHg for KDB and 14.2 ± 2.6 mmHg for TA, *p* = 0.12, and Meds at 12 months were 1.0 ± 1.2 for KDB and 1.4 ± 1.1 for TA *p* = 0.12. The median follow-up was 13.1 (IQR: 11.6–17.1) months for the whole group. A total of four eyes following KDB and two after TA required additional surgery to lower the IOP.

**Conclusion:**

Both KDB and TA with cataract extraction lowered IOP in PEX glaucoma. However, KDB was associated with a higher rate of additional surgical interventions to achieve adequate IOP control.

## Introduction

At present, a growing number of minimally invasive glaucoma surgical (MIGS) are reported to be effective and safe in the setting of randomized clinical trials.^1,2^ Many of these procedures have recently been validated for both efficacy and safety through numerous real-world studies,^3,4^ primarily focusing on primary open-angle glaucoma. These real-world studies offer valuable insights into the applicability of clinical trial results to actual patient care, a crucial aspect for counseling and decision-making.^5^

To the best of our knowledge, this is the first real-world study where the postoperative outcomes in pseudoexfoliation glaucoma (PEX glaucoma) cases treated with clear cornea cataract extraction and intraocular lens implantation combined with ab interno trabeculotomy using the Kahook Dual Blade^®^ Excisional Goniotomy (New World Medical, Rancho Cucamonga, CA, USA, KDB) and trabecular aspiration (TA) are compared. KDB is a disposable surgical blade designed for a single incision, aiming to nearly completely remove the trabecular meshwork over several clock hours with one cut.^6^ TA, as described by Jacobi et al., uses a 400 μm aspiration cannula placed in direct contact with the anterior chamber angle. A vacuum of up to 200 mmHg is applied to treat roughly 270° of the anterior chamber angle.^7^ Both the KDB and TA are non-penetrating glaucoma surgical techniques that do not involve transscleral drainage to the subconjunctival space. Consequently, they typically are not associated with complications commonly reported for filtering procedures, such as hypotony, wound leaks, a flat anterior chamber, choroidal effusion, or hemorrhage.^8^ Furthermore, these techniques preserve conjunctival drainage pathways, potentially facilitating future interventions^9^ and do not need any implant.^6^ Both techniques are used in our clinical praxis in PEX glaucoma patients. However, it remains unclear whether removal of the PEX material by TA alone is as effective as goniotomy with KDB in comparison.

## Methods

A retrospective analysis of all adult PEX glaucoma patients who underwent clear cornea cataract extraction (phaco) combined either with KDB or TA between May 2017 and May 2023 at the Department of Ophthalmology, University Eye Hospital, was performed. Patients who previously underwent glaucoma surgery or were unable to attend follow-up visits were excluded from the study. In all, 113 eyes from 99 patients, with 71 undergoing KDB and 42 TA, were included in this study. A total of 30 KDB patients could be matched to 30 TA patients using propensity score matching based on age, sex, baseline intraocular pressure (IOP), and the number of glaucoma medications.

Demographic data such as age and sex, previous operations, preoperative and postoperative number of topical glaucoma medication, visual acuity, visual field, IOP, complete success and qualified success, operated side, complications, and reoperations were analyzed. IOP was measured using Goldmann applanation tonometry. Three measurements were taken within 2 weeks preoperatively, and an average value was calculated. The complete success was defined as an IOP ⩽ 21 mmHg with at least 20% reduction compared to baseline without eye drops or revisions. The qualified success was defined as meeting the criteria for complete success, but with the use of eye drops. The endpoint for each eye is the last available follow-up/data. Glaucoma patients routinely visit our clinic at 4 weeks, 3 months, 6 months, and 12 months post-operation. This study followed the ethical standards of the Declaration of Helsinki.

## Surgical procedure

Each operation was a combination of cataract surgery and either KDB or TA, performed in a single surgical session. Cataract surgery was conducted first, followed by KDB or TA.

## Cataract surgery

The cataract surgery began with sterile draping and the placement of an eyelid speculum. Paracentesis was made at the 9:00 and 3:00 positions, and a corneal tunnel was created at 12:00 using a 2.8 mm lance. Viscoelastic was then injected, followed by capsulorhexis, hydrodissection, and phacoemulsification. The cortex was aspirated using a bimanual irrigation/aspiration setup, and viscoelastic was injected into the capsular bag and anterior chamber. The lens was implanted into the capsular bag, where it centered well on its own. The viscoelastic was aspirated, and the anterior chamber was filled with BSS. The paracentesis was hydrated, and the tunnel was sealed. Aprocam (cefuroxime) 0.1 ml was injected intracamerally, the eyelid speculum was removed, and a Dexagenta-crème was applied.

## Kahook Dual Blade Excisional Goniotomy

The procedure was performed following the technique described by Francis et al.^10^ A corneal incision was made in the temporal region using a keratome blade, through which a KDB was introduced into the anterior chamber. Under gonioscopic visualization, the KDB was guided across the anterior chamber to penetrate the trabecular meshwork (TM) and enter Schlemm’s canal nasally. Using a gentle lifting motion, the device was rotated within the canal to excise the TM. The excised strip was released by initiating the cut distally and moving in the opposite direction, then extracted with microforceps. Finally, the KDB was removed from the anterior chamber.^10^

## Trabecular aspiration

TA was performed as first described by Jacobi et al. To maintain chamber depth, an irrigation cannula was introduced, while the Jacobi trabecular aspiration cannula (Geuder AG, Heidelberg, Germany) with an outer diameter of 400 μm was utilized in direct contact with the chamber angle, without the need for gonioscopic control. The maximum vacuum of 200 mmHg was applied, and the treatment area covered approximately 270° of the anterior chamber circumference,^7^ made possible by accessing both the 9:00 and 3:00 positions through paracentesis.

The postoperative management consisted of dexamethasone 0.1% six times daily, with a reduction of one drop per week, and ofloxacine eye drops five times daily for 5 days.

## Data analysis

An a priori power analysis was initially conducted to estimate the required sample size. Using a paired *t*-test with a two-tailed significance level of 0.05, a target power of 0.8 and an assumed effect size (Cohen’s *d*) of 0.5, the analysis indicated that a total of 68 eyes (34 in each group) were necessary.

However, to reduce potential confounding factors and ensure balanced baseline characteristics, propensity score matching (PSM) was subsequently performed. This approach prioritized internal validity over maximal statistical power, resulting in two matched groups of 30 eyes each. A post hoc sensitivity analysis was then performed to quantify the impact on detectable effects. This analysis demonstrated that, with the final sample size of 30 eyes per group, the study was powered to detect a minimal difference in mean IOP reduction of approximately 2.4 mmHg (Cohen’s *d* ≈ 0.75).

## Propensity score and optimal pair matching

We used PSM to control for confounding variables. A logistic regression model was fitted with the treatment variable (Surgery), using Age, IOP_preop, Meds_preop, and Sex as covariates to estimate propensity scores.

Matching was performed in RStudio using the package “Matching” with Optimal Pair Matching (method = “optimal”). This method pairs treated (TA, Surgery = 0; Kahook, Surgery = 1) and units by minimizing the total covariate distance across the entire sample. The matching was done 1:1 without replacement, applying a caliper of 0.25 to ensure matches within a narrow propensity score range.

Both treatment and control groups were matched to have the same number of units, ensuring a balanced comparison.

For statistical analysis, BCVA was measured with the Snellen chart and converted to the logarithm of the minimum angle of resolution (logMAR) scale. Categorical variables were presented as absolute and relative frequencies, whereas mean and standard deviation were computed for approximately normally distributed continuous variables, otherwise median and interquartile range. Evaluation of data normality was performed using the D’Agostino and Pearson test. For normally distributed data, a paired *t*-test or ANOVA with Welch’s correction for repeated measures was used. Non-normally distributed continuous data of paired samples were compared by the Wilcoxon signed-rank test. For multiple comparisons, the non-parametric Kruskal–Wallis test and post hoc Dunn’s test were used. All statistical tests were two-sided, and a *p*-value < 0.05 was considered statistically significant. Statistical analysis was performed using GraphPad Prism 10 (Version 10.3.0 (461), GraphPad Software, San Diego, USA) for Mac.

## Results

### Demographics

This study included 113 eyes from 99 patients, with 71 undergoing KDB and 42 TA. A total of 30 KDB patients could be matched to 30 TA patients. Demographics and patients’ characteristics are shown in Table 1. The mean patient age was above 80 years in both groups, with no significant difference between them. In both groups, women accounted for a higher percentage. All patients had pseudoexfoliation glaucoma. None of the included eyes had undergone previous eye surgery. There was no statistically significant difference between the groups regarding the operated side. Both groups had patients with moderate glaucoma based on visual field damage. The median follow-up was 13.1 (IQR: 11.6–17.1) months for the whole group, 13.2 (IQR: 11.9–16.3) months for KDB, and 12.5 (IQR: 8.9–17.2) months for TA (*p* = 0.4). Demographic data of excluded patients are shown in Table 2. These patients differed statistically in terms of age, preoperative IOP, and preoperative topical hypertensive medication, which led to their exclusion from the matched analysis (Table 1).

**Table 1.** Demographic data of matched patients for KDB and TA at baseline.

Demographic and ocular characteristics	KDB (*n* = 30)	TA (*n* = 30)	*p* Value
Baseline			
Age, mean ± SD	80.5 ± 7.3	80.4 ± 7.7	0.94*
Gender: Female, *n* (%)	22 (73.3)	22 (73.3)	1.0**
Eye: Right, *n* (%)	16 (47.1)	20 (58.8)	0.5**
Visual field (MD)	8.6 ± 6.1	6.6 ± 5.4	0.24***
IOP (mmHg), mean ± SD	20.4 ± 7.1	20.0 ± 8.4	0.35*
Topical hypotensive medication, Mean ± SD	2.0 ± 1.2	2.0 ± 1.2	0.78*

* Mann–Whitney test.

** Fisher’s exact test.

*** Unpaired *t*-test with Welch’s correction.

IOP, intraocular pressure; KDB, Kahook Dual Blade^®^ Excisional Goniotomy; TA, trabecular aspiration.

**Table 2.** Demographic data of excluded patients for KDB and TA at baseline.

Demographic and ocular characteristics	KDB (*n* = 41)	TA (*n* = 12)	*p* Value
Baseline			
Age, mean ± SD	73.8 ± 12.3	85.4 ± 3.4	<0.001*
Gender Female, *n* (%)	16 (39.0)	10 (33.3)	0.009**
Eye Right, *n* (%)	15 (36.6)	5 (41.7)	0.75**
Visual field (MD)	8.0 ± 6.2	9.6 ± 8.7	0.67*
IOP (mmHg), mean ± SD	21.7 ± 7.0	18.2 ± 10.3	0.008*
Topical hypotensive medication, mean ± SD	2.8 ± 1.4	0.3 ± 1.2	<0.001*

* Mann–Whitney test.

** Fisher’s exact test.

*** Unpaired t-test with Welch’s correction.

KDB, Kahook Dual Blade^®^ Excisional Goniotomy; TA, Trabecular Aspiration.

### Intraocular pressure

The mean postoperative IOP was reduced at 12 months from 20.4 ± 7.1 at baseline to 13.5 ± 3.6 mmHg for KDB and from 20.0 ± 8.4 at baseline to 14.2 ± 2.6 mmHg for TA, respectively. The change in IOP for KDB −5.6 mmHg (*p* < 0.01) and for TA −5.3 mmHg (*p* < 0.01).

**Figure 1.**
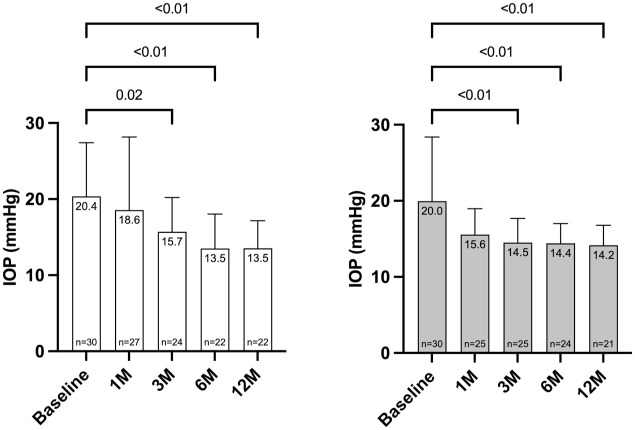
IOP at baseline and at every follow-up for KDB and TA. IOP in mmHg indicates intraocular pressure; 1M: 1 month; 3M: 3 months; 6M: 6 months; 12M: 12 months.

The mean IOP for KDB was 18.6 ± 9.6 mmHg (−1.8 mmHg, 8.8%) at 1 month (*p* = 0.04), 15.7 ± 4.5 mmHg (−4.7 mmHg, 23%) at 3 months (*p* < 0.01), 13.5 ± 4.5 mmHg (−6.9 mmHg, 33.8%) at 6 months (*p* < 0.01), and 13.5 ± 3.6 mmHg (−6.9 mmHg, 33.8%) at 12 months (*P* < 0.01) (Figure 1).

The mean IOP for TA was 15.6 ± 3.4 mmHg (−4.4 mmHg, 22%) at 1 month (*p* < 0.01), 14.5 ± 3.2 mmHg (−5.5 mmHg, 27.5%) at 3 months (*p* < 0.01), 14.4 ± 2.6 mmHg (−5.6 mmHg, 28%) at 6 months (*p* < 0.01), and 14.2 ± 2.6 mmHg (−5.8 mmHg, 28%) at 12 months (*p* < 0.01; Figure 1).

### Topical glaucoma medication

**Figure 2.**
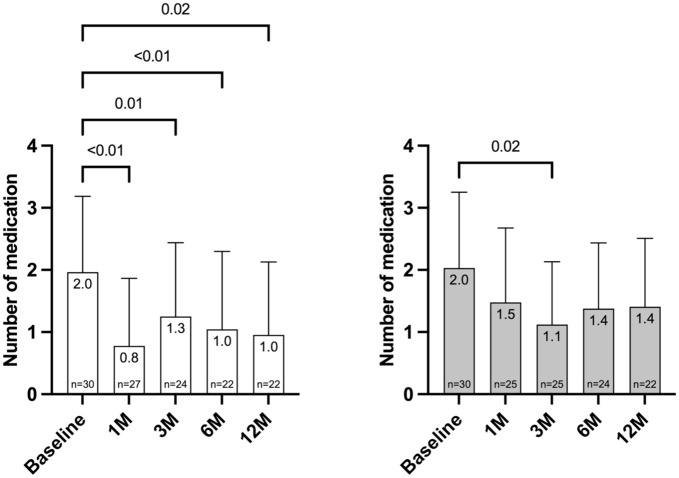
Topical glaucoma medication at baseline and postoperatively for KDB and TA; IOP in mmHg indicates intraocular pressure; 1M: 1 month; 3M: 3 months; 6M: 6 months; 12M: 12 months. Table 2.

The number of topical glaucoma medications decreased from 2.0 ± 1.2 at baseline to 1.0 ± 1.2 (−1.0, 50%) at 12 months for KDB and 2.0 ± 1.2 at baseline to 1.4 ± 1.1 (–0.6, 30%) for TA. However, neither of these changes was statistically significant (*p* = 0.12). Topical glaucoma medication for KDB was 0.8 ± 1.1 (−1.2, 60%) after 1 month (*p* < 0.01), 1.3 ± 1.2 (−0.7, 35%) after 3 months (*p* = 0.01), 1.1 ± 1.3 (−0.9, 45%) after 6 months (*p* *⩽* 0.01), and 1.0 ± 1.2 (−1.0, 50%) after 12 months (*p* = 0.02) (Figure 2). Antiglaucoma medication was for TA 1.5 ± 1.2 (−0.5, 25%) after 1 month, 1.1 ± 1.0 (−0.9, 45%) after 3 months (*p* = 0.02), 1.4 ± 1.1 (−0.6, 30%) after 6 months, and 1.4 ± 1.1 (−0.6, 30%) after 12 months (Figure 2).

**Figure 3.**
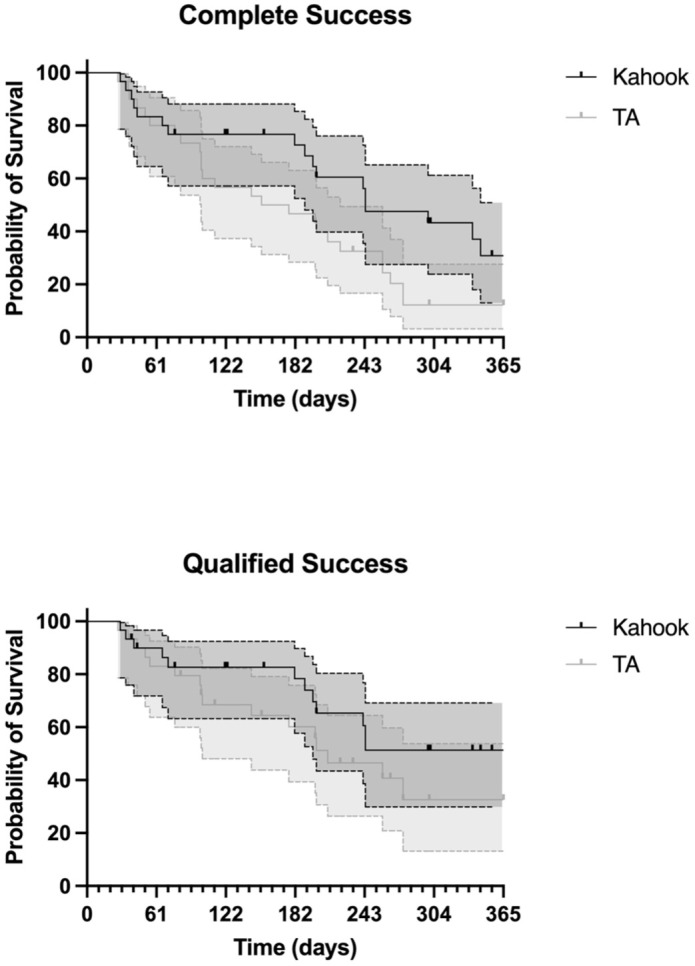
Complete and qualified success for KDB and TA.

### Complete and qualified success

After 12 months, the complete success rate was 30.8% for KDB and 12.2% for TA. The qualified success rates were 51.3% and 32.6%, respectively. No statistically significant difference in success rates was observed between the two groups.

The survival curves for complete success in the KDB and TA were compared using the Log-rank (Mantel–Cox) test, which revealed no significant difference in survival distributions (χ² = 2.71, df = 1, *p* = 0.10). Nonetheless, the median survival time was longer in the KDB (244 days) than in the TA (165 days) (Figure 3).

For qualified success, the Log-rank analysis likewise indicated no significant difference between the two groups (χ² = 0.62, df = 1, *p* = 0.43). However, the median survival time was again greater for the KDB (370 days) compared to TA (211 days) (Figure 3).

### Complications and reoperations

In the KDB group, four eyes (13.3%) required additional surgery to lower IOP: two underwent trabeculectomy, one XEN-Implantation, and one cyclophotocoagulation. After TA, two eyes needed the following operation (6.7%), one received trabeculectomy, and another underwent vitrectomy due to persistent vitreous hemorrhage. There were no statistically significant differences concerning reoperations, *p* = 0.67. Patients who underwent a glaucoma re-operation had a median follow-up of 14.2 (IQR 3.5–19.9) months, with IOP of 15 ± 2.4 mmHg while on 1.8 ± 1.8 glaucoma medications. Three of these patients required a third glaucoma operation (1 TE, 2 needlings) to achieve IOP control.

## Discussion

Our study showed that both cataract extraction with KDB and TA effectively lowered IOP in pseudoexfoliation glaucoma. These findings align favorably with outcomes observed in other studies.^7,11^

Regarding PEX glaucoma, elevated IOP is primarily caused by increased outflow resistance within the TM.^12^ This resistance results from blockage of the outflow channels by PEX material, followed by degenerative changes in the wall of Schlemm’s canal.^12^ Consequently, the mechanisms of both KDB and TA are plausible approaches for reducing IOP in PEX glaucoma patients. TA works by aspirating the PEX material, while KDB removes a portion (up to 120°) of the altered TM, thereby unroofing Schlemm’s canal. Such differing surgical approaches can result in distinct postoperative success rates.

The mean postoperative IOP in our study was reduced from 20.0 ± 8.4 mmHg at baseline to 14.2 ± 2.6 mmHg after 12 months for TA (*p* < 0.01). This is consistent with other studies on TA, which have demonstrated effective IOP reduction. In the Jacobi et al.’s prospective study with TA of 12 PEX glaucoma patients, where IOP dropped from a notably high preoperative mean IOP of 37.4 mmHg to 18.3 mmHg after 15 months postoperatively.^7^ Similarly, in the retrospective study of Grüb et al. on 17 eyes, the IOP reduced from 26.8 mmHg before surgical intervention to 19.2 mm Hg after 180 days.^13^ Another retrospective study involving a total of 55 PEX glaucoma patients who received either phaco with TA or phaco with trabeculotomy ab interno with Trabectome showed a reduction from 22.2 ± 6.3 mmHg to 17.1 ± 4.0 mmHg after 1 year (*p* = 0.02) in the TA group.^14^ The preoperative IOP level was similar to this in our study. In a recent study by Prokosch et al. on PEX glaucoma patients with TA or phaco with TA, IOP decreased from 18.0 to 15 mmHg at 36 months, with stable glaucoma medication use.^11^ Klamann et al. also reported a mean IOP reduction from 22.2 to 17.1 mmHg 1 year after phaco-TA.^14^

In our study, after KDB, IOP decreased from 20.4 ± 7.1 mmHg at baseline to 13.5 ± 3.6 mmHg after 1 year. Our data correspond with findings from several prospective and retrospective reports on the outcomes of KDB.^15–18^ For instance, in a retrospective study of 205 eyes in total, IOP decreased from 24.7 ± 7.98 mmHg to 17.6 ± 4.80 mmHg after KDB in 90/205 PEX glaucoma eyes.^15^ Greenwood et al. reported in the multicenter study, a 26% reduction (from 17.4 ± 5.2 mmHg to 12.8 ± 2.6 mmHg) after 6 months with phaco-KDB.^15,16^ However, there were only 3% of PEX glaucoma patients within the study group. Dorairaj et al. reached a comparable 26% reduction in IOP following phako-KDB, decreasing from 16.8 ± 0.6 mmHg to 12.4 ± 0.3 mmHg at 12 months.^15–17^ However, the majority of patients in their cohort had primary open-angle glaucoma (84.6%), while only 3.9% were diagnosed with PEX glaucoma. In comparison, the IOP change was −5.6 mmHg (27%) in our study.

Studies by Wakil et al. and Sieck et al. demonstrated significant IOP reduction with phaco-KDB and KDB as a standalone procedure.^19,20^ Wakil et al. reported a decrease from 16.5 ± 5.0 mmHg to 14.1 ± 3.9 mmHg for the combined phako-KDB approach and from 24.3 ± 9.1 mmHg to 16.9 ± 7.6 mmHg for standalone KDB after 12 months, with similar results observed at 18 months.^17,19^ Comparable findings were reported in Sieck et al. ’s study,^19,20^ which included 16.8% of patients with PEX glaucoma.^19,20^

The randomized clinical trial COMPASS and HORIZON reported that Phaco as a standalone procedure reduces IOP itself in open-angle glaucoma by approximately 5 mmHg after 2 years.^21,22^

According to our results, the number of topical glaucoma medications at 12 months decreased for KDB and TA. However, for both interventions, these changes were not statistically significant (*p* > 0.99). Postoperative medication reductions reported in various retrospective studies are difficult to compare due to the lack of a standardized protocol for discontinuing medications. While some surgeons stop all medications immediately after surgery and reintroduce them as needed, others adopt a more conservative approach, gradually discontinuing medications based on IOP measurements at follow-up visits. The postoperative management with medications is often not detailed described. Jacobi et al. reported a reduction of glaucoma medication from 4.3 to 1.4 after 15 months after TA by PEX glaucoma^21^ and Grüb et al. from 3.1 to 1.0 after 180 days.^13^

Greenwood et al. found a decrease from 1.6 ± 1.3 to 0.9 ± 1.0 at 6 months after KDB,^15,16^ and Dorairaj et al. from 1.6 ± 0.2 to 0.8 ± 0.1 at month 12, signifying a 50.0% reduction.^17^ Similar reductions were noted in studies with phako-KDB by Falkenberry et al. on open-angle glaucoma patients^18^ and Ventura-Abreu et al. on open-angle glaucoma or ocular hypertension,^6^ while Klamman et al. observed a non-significant decrease from 2.3 to 2.0 on PEX glaucoma patients.^14^

Both groups had relatively low rates of complete and qualified success. To date, most MIGS studies do not report standardized success criteria, leaving little comparative data available. Furthermore, thresholds like a 20% IOP reduction—especially when preoperative IOP is already below 20 mmHg—are rarely achievable in MIGS. Lastly, the primary objectives of MIGS procedures are to lower IOP and reduce medication burden, rather than eliminate eye drops altogether.

As in the study by Jacobi et al.^23^ or Ventura-Abreu et al.,^6^ our study found no major complications during or after surgery. However, five eyes, four after KDB, and one after TA developed IOP hypertension requiring further surgery. One TA patient experienced persistent vitreous hemorrhage and underwent vitrectomy.

Our study has several limitations, including its retrospective design and small sample size. Due to the retrospective nature of the study, washout IOP was not recorded prior to surgery, which may have underestimated the procedure’s effectiveness in lowering IOP. In addition, a longer follow-up period would be valuable, as IOP may increase over time. At the 12-month endpoint, IOP outcomes reflect only those responders who did not require secondary surgery. Moreover, our data collection focused exclusively on complications that necessitated reoperation, while minor events such as microhyphema were not documented.

Despite these limitations, this is, to the best of our knowledge, the first study comparing combined phaco with either TA or KDB. The study’s strengths include the use of PSM and optimal pair matching, which reduce selection bias and control for confounding variables. The utilization of PSM pairs individuals with similar characteristics across treatment groups, while optimal pair matching reduces covariate differences between pairs, resulting in a more accurate and balanced analysis. We excluded patients who differed significantly in age, preoperative IOP, and preoperative topical antihypertensive medication, as including them could have led to misleading conclusions regarding postoperative outcomes. Further validation will require prospective studies with larger patient cohorts.

## Conclusion

Both cataract extraction with KDB and TA effectively lower IOP in patients with PEX glaucoma. However, KDB was associated with a higher rate of additional surgical interventions to achieve adequate IOP control.

## Declarations

**Ethics approval and consent to participate:** The study was approved by the Ethics Committee at the Medical Faculty of Heinrich Heine University Düsseldorf (No. 2024-27750). The requirement of informed consent was waived by the Ethics Committee.

**Consent for publication:** Not applicable.

**Availability of data and materials:** The datasets generated during and/or analyzed during the current study are available from the corresponding author on reasonable request. The partial results were presented as a poster at the EGS Conference in Dublin in 2024.
